# Resistance to Germline RNA Interference in a *Caenorhabditis elegans* Wild Isolate Exhibits Complexity and Nonadditivity

**DOI:** 10.1534/g3.113.005785

**Published:** 2013-06-01

**Authors:** Daniel A. Pollard, Matthew V. Rockman

**Affiliations:** Department of Biology, New York University, New York, NY 10003

**Keywords:** QTL, epistasis, RNAi

## Abstract

Resolving the genetic complexity of heritable phenotypic variation is fundamental to understanding the mechanisms of evolution and the etiology of human disease. Trait variation among isolates from genetically efficient model organisms offers the opportunity to dissect genetic architectures and identify the molecular mechanisms of causation. Here we present a genetic analysis of loss of sensitivity to gene knockdown via exogenous RNA interference in the germline of a wild isolate of the roundworm *Caenorhabditis elegans*. We find that the loss of RNA interference sensitivity in the wild isolate CB4856 is recessive to the sensitivity of the lab strain N2. A cross of the strains produced F2 with intermediate sensitivities, and the segregation of the trait among F2s strongly deviated from a single locus recessive allele expectation. Linkage analysis in recombinant inbred lines derived from CB4856 and N2 identified a single significant locus on chromosome I that includes the argonaute gene *ppw-1*. The alleles for *ppw-1* were unable to explain the sensitivity of 18 (12.1%) of the recombinant inbred lines. Complementation tests and F2 segregation analysis of these recombinant inbred lines revealed cases of complex epistatic suppression and enhancement of the effects of *ppw-1*. We conclude that the variation in RNA interference sensitivity between CB4856 and N2 likely involves the nonadditive interactions of eight or more genes in addition to *ppw-1*.

The genetic complexity of natural phenotypic variation in populations is of central importance for understanding the etiology of human disease and the mechanisms of evolution. The tractability of mapping highly penetrant monogenic traits, such as cystic fibrosis in humans (Wainwright *et al.* 1985), has facilitated many important discoveries. However, most common heritable phenotypic variation is the result of the action of multiple genetic variants and environmental variables. The contribution of each genetic variant to the total trait variance is typically unequal and can depend on the state of the other contributing loci [*i.e.*, epistasis (Phillips 2008)]. Different combinations of alleles of causative loci result in a distribution of trait values, even when environmental variables are held constant. Complex traits are studied using marker association with trait values in populations or linkage analysis in experimental crosses. Such approaches have dissected traits as genetically simple as the digenic oxygen and carbon dioxide−avoidance behavior in nematode worms (McGrath *et al.* 2009) and as complex as the highly polygenic liability to schizophrenia in humans (Lee *et al.* 2012). Such examples make clear that phenotypic variation can be the result of an enormous range of numbers of loci. However, for most traits, it remains unclear how genetically complex they are, how the trait variation is distributed across the loci, and the degree to which loci act additively or epistatically.

Complex trait analysis in model systems with powerful and efficient genetic tools, such as the roundworm *Caenorhabditis elegans*, has the potential to shed light on the distribution of alleles and allelic interactions that produce heritable phenotypic variation. Wild isolate strains of *C. elegans* exhibit diversity in many traits, from biochemical (Tijsterman *et al.* 2002; Rockman *et al.* 2010) to physiological (Palopoli *et al.* 2008) to behavioral (McGrath *et al.* 2009; Bendesky *et al.* 2011). In recent quantitative trait locus (QTL) mapping studies in *C. elegans* investigators have successfully identified causative genetic variants, such as the regulatory variant in the *tyra-3* gene affecting exploration-exploitation decision making (Bendesky *et al.* 2011).

RNA interference (RNAi), the process by which exogenous double-stranded RNA (dsRNA) leads to degradation of complementary endogenous RNA, was first discovered and characterized in *C. elegans* (Fire *et al.* 1998). Fortuitously, the laboratory strain N2, which is used for the vast majority of research in *C. elegans*, is highly sensitive to RNAi whereas other strains in the population vary greatly in their RNAi responses (Tijsterman *et al.* 2002; Felix 2008; Elvin *et al.* 2011; Felix *et al.* 2011). The molecular mechanisms underlying RNAi variation are of great importance because of the widespread use of RNAi as a powerful reverse-genetics technique and because of the shared machinery between RNAi and other small RNA biogenesis pathways (*e.g.*, microRNAs).

The RNAi pathway consists of two core steps that are inferred to have originated in the stem eukaryote and subsequently diversified (Shabalina and Koonin 2008). In the first step of the RNAi pathway, dsRNA is cleaved by the protein Dicer into 21−23 nucleotide double-stranded, small interfering RNAs. Second, the small interfering RNAs are incorporated into the RNA-induced silencing complex and complementary RNAs are cleaved by argonaute, the complex's catalytic protein. Unlike most eukaryotes, *C. elegans* responds to RNAi systemically. Because dsRNA can quickly spread to cells throughout the body, RNAi can be initiated through exposure, typically by feeding with bacteria expressing dsRNA or by soaking in dsRNA.

Strains of *C. elegans* exhibit variation in RNAi sensitivity in either the germline or the soma. None of the wild strains that have been examined has exhibited loss of RNAi in both the germline and soma (Tijsterman *et al.* 2002; Felix *et al.* 2011), suggesting mechanisms independent of the systemic response. The molecular mechanisms for somatic RNAi variation have not been studied, although somatic RNAi variation has been linked to variation in viral replication during infection (Felix *et al.* 2011). One wild strain isolated from a pineapple field in Hawaii, CB4856 (Hodgkin and Doniach 1997), exhibits dramatic loss of germline RNAi sensitivity (Tijsterman *et al.* 2002; Elvin *et al.* 2011). Genetic analysis suggests the loss of germline RNAi sensitivity in CB4856 has a complex genetic basis and is associated with polymorphisms in the argonaute encoding gene, *ppw-1* (Tijsterman *et al.* 2002; Elvin *et al.* 2011).

Here we describe our analysis of the genetic basis for variation in germline RNAi sensitivity between the *C. elegans* strains N2 and CB4856. We use feeding RNAi of bacteria expressing dsRNA targeting the essential maternal-effect gene *par-1*, an established assay for the efficacy of germline RNAi (Tijsterman *et al.* 2002). Using classical and quantitative genetic approaches, we find evidence that the difference in germline RNAi sensitivity between N2 and CB4856 is due to the action of many genes with both additive and epistatic effects.

## Materials and Methods

### Bacteria strains

For growth and maintenance, worms were fed OP50-1. For *par-1* RNAi treatment, worms were fed HT115(DE3) bacteria with *par-1* polymerase chain reaction (PCR) products cloned into the Timmons and Fire feeding vector (L4440) from the Ahringer library (GeneService ID V-9E06) (Kamath and Ahringer 2003). For untreated RNAi controls, worms were fed HT115(DE3) bacteria with the empty feeding vector (L4440) from the Ahringer library.

### *C. elegans* strains

Wild-type strains N2 (standard lab strain) and CB4856 (Hawaiian wild isolate) were provided by the CGC, which is funded by the National Institutes of Health Office of Research Infrastructure Programs (P40 OD010440).

Alleles *ppw-1(pk1425)* I, a deletion with recessive loss of germline RNAi sensitivity, and *lon-2(e678)* X, which confers a recessive long phenotype, were also attained from the CGC, each in the N2 background. Allele *e678* was used in crosses to confirm outcrossed F1. We generated a double mutant of alleles *pk1425* and *e678* by crossing and using PCR to genotype *pk1425* (primers: CCGGTGTTTGCGTACTTTTT, AAAAACCGACACCCTTGAGA). We introgressed *e678* into the CB4856 genetic background by 10 generations of backcrossing. No interaction was detected between *e678* and RNAi phenotypes.

Recombinant inbred advance intercross lines (RIAILs), QX1 through QX237, were generated previously (Rockman and Kruglyak 2009) from a cross of N2 and CB4856. Each RIAIL was genotyped at 1455 single nucleotide polymorphic markers throughout the genome. See (Rockman and Kruglyak 2009) for more details.

### Feeding RNAi assay

The assay assesses the effect of feeding a worm bacteria expressing *par-1* dsRNA. The effect of the RNAi treatment is not observed in the fed worm but instead is read out by the degree of induced embryonic lethality in the worm’s offspring.

*par-1* and empty vector (control) bacteria were streaked out on Amp/Tet LB plates from frozen stocks and grown overnight at 37°. A single colony was picked and used to inoculate an LB + Amp overnight liquid culture. Then, 50 µL of culture was added as a contiguous circular lawn, about 1 cm in diameter, to 6 cm nematode growth media + 1 mM isopropyl β-D-thiogalactoside + 25 µg/mL carbenicillin plates. Plates were left at room temperature overnight to allow the bacterial lawn to grow.

For at least two generations before experiments and during experiments, *C. elegans* strains were kept well fed at 20° (except where noted). To begin an experiment, L4 hermaphrodites were transferred to RNAi bacteria plates (either *par-1* or *L4440*) and then transferred to fresh RNAi bacteria plates 24 hr later. Eighteen hours later (42 hr after L4), when the worms were gravid adults, worms were transferred to fresh RNAi bacteria plates for a 6-hr timed embryo lay. After the 6-hr timed lay adults were removed and discarded. A minimum of 12 hr later, dead fertilized embryos and hatched larvae were counted on each plate.

### Induced embryonic lethality

Counts of dead embryos and hatched larvae were used to calculate the fraction of laid embryos that arrest, *i.e.*, the embryonic lethality of the RNAi treatment for the strain. We isolated the specific embryonic lethality effects of *par-1* RNAi treatment from any background embryonic lethality using the embryonic lethality on the *L4440* (empty vector) RNAi plates.

$$\mathrm{InducedLethality}=\frac{\frac{DE_{par-1}}{DE_{par-1}+HL_{par-1}}-\frac{DE_{L4440}}{DE_{L4440}+HL_{L4440}}}{1-\frac{DE_{L4440}}{DE_{L4440}+HL_{L4440}}}$$

where *DE* is the count of dead fertilized embryos and *HL* is the count of hatched larvae.

### Dominance

Dominance was calculated as (Falconer 1989):

$$d=\frac{Hybrid-MidParent}{\left|ParentA-MidParent\right|}$$

### F2 segregation

N2 males were crossed with QG145 (Lon CB4856) hermaphrodites, non-Lon F1 hermaphrodites were singled, and 181 F2 were subject to the feeding RNAi assay starting at L4 stage of development. Similarly, for the segregation of *ppw-1(pk1425)* I, N2 males were crossed with QG146 [Lon *ppw-1(pk1425)* I] hermaphrodites, non-Lon F1 hermaphrodites were singled, and 197 F2 were subject to the feeding RNAi assay.

Segregation analysis of *ppw-1(CB4856)* in the N2 × CB4856 cross is complicated by its linkage to the *zeel-1/peel-1* interval, a locus deleted in CB4856. Zygotic *zeel-1* expression is required to prevent embryonic lethality induced by paternal-effect *peel-1* (Seidel *et al.* 2008). The *zeel-1/peel-1* locus and *ppw-1* are separated by 7.47 cM, according to the WormBase (http://www.wormbase.org) interpolated genetic map. We used this genetic distance to estimate the expected frequencies of F2 phenotype classes, as detailed in Supporting Information, Figure S1, under the assumption that the CB4856 allele of *ppw-1* confers recessive resistance to *par-1* RNAi. We allowed the penetrance of paternal *peel-1*-induced lethality among embryos lacking *zeel-1* to be a variable, given that its penetrance in hermaphrodite self-progeny is age-dependent (Seidel *et al.* 2011). We estimated penetrance directly from embryonic lethality in crosses of N2 and CB4856 (see Table S1).

### Linkage mapping

We used the R programming language package Rqtl (Broman *et al.* 2003) for linkage analysis. Using 149 RIAIL induced embryonic lethality levels and genotypes (see Table S2 and File S1), we performed nonparametric interval mapping with 1-cM spacing. The genome-wide significance was estimated using 1000 permutations of the induced embryonic lethality assignments. To incorporate the *ppw-1*-flanking marker interval as a covariate, we used a parametric normal model for the interval mapping.

### Genotyping *ppw-1* alleles

We distinguished N2 and CB4856 alleles of *ppw-1* by sequencing through chromosome 1 position 4,187,632, which is the position of a single nucleotide indel polymorphism. A 5-kb fragment was amplified with PCR from genomic DNA preparations of N2, CB4856, QX217, and QX222 using primers targeting chromosome 1, positions 4,186,070 to 4,191,078 (CCGGTGTTTGCGTACTTTTT; AAAAACCGACACCCTTGAGA). The large PCR fragment is necessary to avoid amplifying the close paralog *sago-2*. A sequencing primer at chromosome 1 position 4,187,022 (TGAGGTGAATTCGATCAAGC) was used to sequence through the indel polymorphism.

## Results

### N2’s sensitivity to *par-1* RNAi is dominant to CB4856s insensitivity

To estimate the dominance of N2’s high sensitivity to germline RNAi over CB4856’s low sensitivity, we measured induced lethality from *par-1* RNAi treatment (see *Materials and Methods*) in individual N2, CB4856 and F1 (from a cross of N2 males and CB4856 hermaphrodites) (Table S1). N2 showed consistent 100% induced lethality as expected. CB4856 showed 0% induced lethality in six individuals and 0.1–1.5% induced lethality in two individuals. The F1 showed 100% induced lethality in eight individuals and 91–96% induced lethality in two individuals. We estimate that N2’s high induced lethality has a dominance of 0.982 over CB4856s low induced lethality (see *Materials and Methods*).

### *ppw-1* alone cannot explain segregation of *par-1* RNAi sensitivity

To evaluate the complexity of the difference in effects of *par-1* RNAi on N2 and CB4856, we looked at the segregation pattern of induced lethality from a cross of the strains. We crossed N2 males with CB4856 hermaphrodites, selfed the F1s, and tested 181 F2. We classified F2 into the four phenotypic categories: full sensitivity (100% lethality), high sensitivity (75–99% lethality), intermediate sensitivity (25–75% lethality), and low sensitivity (<25% lethality). We note that this classification is based on embryonic lethality after *par-1* RNAi treatment alone, without a control for background levels of embryonic lethality.

Across the 181 F2, we found that 109 (60.2%) showed full sensitivity, 22 (12.2%) showed high sensitivity, 12 (6.6%) showed intermediate sensitivity, and 38 (21.0%) showed low sensitivity. The authors of a previous study reported 47% F2 with full sensitivity and 53% F2 with lower sensitivities for this cross (Tijsterman *et al.* 2002). Our results are similar, though significantly different (χ^2^ test, *P* < 0.01).

As a control to isolate the effects of *ppw-1* from all other segregating effects, we crossed NL3511 (*ppw-1* deletion in N2 background with low sensitivity) with N2 and measured lethality after *par-1* RNAi treatment in 197 F2. Sensitivity segregated 3:1, full sensitivity to low sensitivity, with all but 1 of 197 F2 showing either full sensitivity or low sensitivity (data not shown).

The presence of a large (18.8%) intermediate class of N2xCB4856 F2 that is neither fully sensitive (like N2) nor highly insensitive (like CB4856) excludes the hypothesis that *ppw-1* alone could explain both the insensitivity of CB4856 and the segregation results. We observe more F2 with less than full sensitivity (39.8%) than expected from segregation of *ppw-1* alone (25%), however, this expectation fails to take into account what we know about the chromosomal context of *ppw-1*.

*ppw-1* is linked to the *zeel-1*/*peel-1* incompatibility locus (Seidel *et al.* 2008), which greatly changes the expected segregation pattern in a cross of N2 and CB4856. Worms homozygous for the CB4856 allele of *zeel-1* often die (or suffer developmental delays and deformities) in the presence of the paternally deposited toxin (PEEL-1), whereas worms containing at least one N2 allele of *zeel-l* are unaffected by the toxin (Seidel *et al.* 2008, 2011). All F1 male gametes will deposit PEEL-1, and therefore the *zeel-1* genotype of F2 hermaphrodites will determine if they are susceptible to PEEL-1 (see Figure S1). *zeel-1* and *ppw-1* are 7.47 cM apart on chromosome 1 (http://www.wormbase.org), so only a small fraction of gametes with recombination between *zeel-1* and *ppw-1* would be expected to produce F2 that are alive and germline RNAi insensitive due to *ppw-1*. To estimate the expected embryonic lethality levels in F2 due to *ppw-1* and *zeel-1/peel-1*, we measured the penetrance of embryonic lethality from F1 of crosses of N2 and CB4856 (Table S1). We find a PEEL-1 toxin penetrance of 26%, which is lower than the 70–90% expected for age-matched worms from Seidel *et al*. but consistent with older worms from their study (Seidel *et al.* 2011). Assuming a PEEL-1 penetrance of 26%, we would expect 79.2% of F2s to exhibit total embryonic lethality, 17.1% to show no lethality, and 3.7% to show low lethality (Figure S1A). Although these expectations can explain the observed proportion of F2s with low sensitivity, they fail to explain the proportion with high sensitivity (60.2%; χ^2^ test, *P* < 10^−9^). Greater PEEL-1 penetrance values lead to more total embryonic lethality F2s and fewer no/low lethality F2s (Figure S1B), which produces even stronger expected deviations from the observed F2 proportions. Therefore we can reject *ppw-1* as the sole genetic explanation for our *par-1* RNAi observations.

### Mapping lethality reveals large-effect QTL

To identify the number and effect sizes of the loci contributing to the difference in germline RNAi sensitivity between N2 and CB4856, we used induced lethality as a quantitative trait and mapped causal variants using linkage analysis. We measured induced lethality in 149 RIAILs derived from N2 and CB4856 (Rockman and Kruglyak 2009) (see Table S2 and *Materials and Methods*).

**Figure 1:**
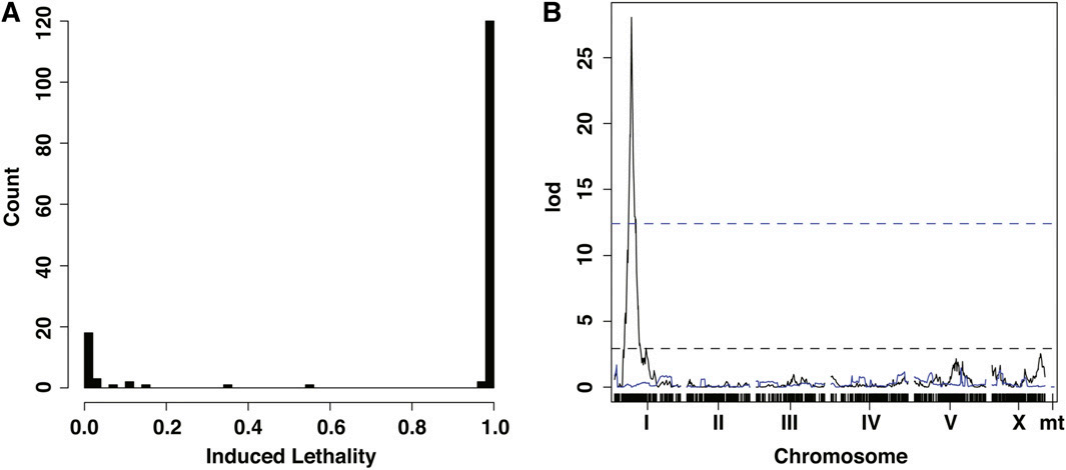
(A) Distribution of *par-1*-induced lethalities across 149 N2 × CB4856 RIAILs. (B) Log Odds (lod) score of nonparametric interval mapping with 1-cM spacing of *par-1*−induced lethality and each of 1455 markers across the five autosomes, the X chromosome, and the mitochondrial genome (black). Parametric normal interval mapping with marker at position 4,175,488 on chromosome 1 as a covariate (blue). Horizontal dashed line is 5% *P*-value from 1000 permutations of induced lethality assignments.

One hundred twenty (80.5%) of the 149 RIAILs had 100% induced lethality, 11 (7.4%) RIAILs had 0% induced lethality, and 18 (12.1%) RIAILs had induced lethality between 0% and 100% (see Figure 1A and Table S2). Most intermediate-induced lethality RIAILs showed low induced lethality, with only 3 of 18 >50% and only 7 of 18 >10% (see Table 1 and Table S2). The presence of an intermediate class of RIAILs further supports the hypothesis that loci in addition to *ppw-1* are contributing to induced lethality variation in response to *par-1* RNAi.

**Table 1:** RIAILs with intermediate levels of induced lethality

Strain	Mean Induced Lethality (n = 2)	*ppw-1* 5′ Marker	*ppw-1* 3′ Marker
QX57	0.00098	CB4856	CB4856
QX56	0.00279	CB4856	CB4856
QX145	0.00380	CB4856	CB4856
QX236	0.00403	CB4856	CB4856
QX113	0.00420	CB4856	CB4856
QX169	0.00549	CB4856	CB4856
QX127	0.01111	CB4856	CB4856
QX115	0.02231	CB4856	CB4856
QX1	0.02427	CB4856	CB4856
QX218	0.0324	CB4856	CB4856
QX158	0.07819	CB4856	CB4856
QX7	0.11031	CB4856	CB4856
QX168	0.11475	CB4856	CB4856
QX217	0.15780	N2	N2
QX24	0.34959	N2	N2
QX64	0.55530	N2	N2
QX13	0.97357	N2	N2
QX222	0.97357	CB4856	CB4856

Genome-wide linkage mapping of induced lethality in the RIAILs produced a single highly significant QTL on chromosome 1 (see Figure 1B), with several nonrecombinant markers at its peak (3,989,631−4,175,488). The 1.5 Log Odds (lod) confidence interval spans 347.3 kb (3,890,036−4,237,066) containing 77 genes. A scan of these genes revealed that *ppw-1* lies within the interval (4,185,062−4,189,930) and is the only obvious candidate causal locus. When we considered only strains homozygous for the N2 allele at the marker closest to *ppw-1* (4,175,488), or when we included this marker as a covariate and repeated the linkage mapping (see Figure 1B), no significant QTL were recovered from the secondary scan.

The induced lethality in the RIAILs is highly predictive of the genotype of the markers flanking the *ppw-1* locus. Of the 120 RIAILs with 100% induced lethality, 117 are flanked by N2 markers, and the other three are flanked by one N2 marker and one CB4856 marker, presumably cases of recombination between *ppw-1* and the CB4856 marker. All 11 RIAILs with 0% induced lethality are flanked by CB4856 markers. Thus, the interval mapping produced a QTL containing *ppw-1* that can explain variation in *par-1* RNAi response in 131 of 149 (87.9%) RIAILs. We next focused on the variation in the 18 (12.1%) RIAILs that could not be fully explained by the inferred *ppw-1* genotype.

### Intermediate lethality recombinants suggest additive and epistatic interactions with *ppw-1*

We hypothesized that RIAILs with intermediate lethality levels would have the N2 allele for *ppw-1* based on the assumption that homozygosity for the CB4856 allele of *ppw-1* would confer near-total insensitivity (as seen in CB4856 and the strain NL3511 which contains the *ppw-1* deletion allele pk1425 in the N2 background). Quite to the contrary, only four of the 18 RIAILs with intermediate induced-lethality levels have N2 *ppw-1* flanking markers (see Table 1 and Table S2). The observation, that 14 RIAILs have intermediate induced lethality levels and yet have CB4856 *ppw-1* flanking markers suggests that additional loci contribute to both decreases *and* increases in induced lethality and that the increasing effects are background dependent. Of the 14 RIAILs with intermediate induced lethality levels and CB4856 *ppw-1* flanking markers, 13 have induced lethality <12%, consistent with small effect interacting loci. RIAIL QX222, however, has an induced lethality of 97.4%, implying an epistatic interaction that almost completely reverses the effect of its CB4856 *ppw-1* allele. The four RIAILs with intermediate induced-lethality levels and N2 *ppw-1* flanking markers (QX13, QX24, QX64, and QX217) range in induced lethality levels from 16 to 97%, which is consistent with multiple loci contributing to variation in lethality, independent of *ppw-1*. Thus, several additional loci are potentially interacting additively and epistatically with *ppw-1* to generate the variation observed in the intermediate induced-lethality RIAILs.

### Crosses between recombinants and parents reveal *ppw*-1−independent variation and *ppw-1* repressor variation

Given the evidence for additional loci acting either additively or epistatically to generate intermediate induced lethality levels in the RIAILs, we sought to understand which parent the alleles affecting sensitivity derive from, whether the alleles are dominant, and whether the alleles are independent of *ppw-1*. Focusing on two RIAILs, QX217 and QX222, we looked at induced lethality in F1 from crosses of the RIAILs with N2, CB4856, and NL3511 (*ppw-1* deletion in N2 background with low sensitivity).

**Figure 2:**
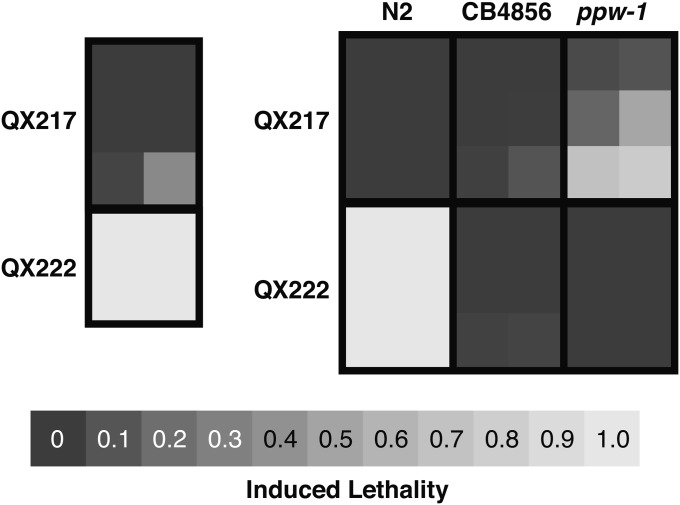
Induced lethality in RIAILs QX217 (*ppw-1(N2)*) and QX222 (*ppw-1(CB4856)*), and F1 from crosses with N2, CB4856, and *ppw-1(pk1425)*. Induced lethality for individual *par-1* RNAi trials are depicted as squares within black rectangles.

QX217 contains the N2 allele of *ppw-1*, confirmed by direct sequencing, and yet it is highly insensitive to *par-1* RNAi treatment (see Table 1 and Table S2). We therefore expected QX217 to contain at least one allele from CB4856 that causes insensitivity. F1 from crosses of QX217 with each parent strain (N2 and CB4856) have very low induced lethality (see Figure 2). The dominance of QX217’s insensitivity over N2’s sensitivity distinguishes it from the two alleles of *ppw-1* we have examined (CB4856 and pk1425), which are both recessive to N2. Furthermore, CB4856s insensitivity is highly recessive to N2’s sensitivity, so QX217’s dominant insensitivity is not strictly derived from CB4856. We therefore infer that QX217 contains a combination of alleles from both N2 and CB4856 that generate its dominant insensitivity. The greater levels of induced lethality in QX217×NL3511 F1 relative to QX217×N2 F1 imply a counterintuitive dependence between *ppw-1* and the insensitivity alleles carried by QX217. Regardless, it is clear that multiple alleles from both N2 and CB4856 contribute to variation in *par-1* RNAi sensitivity.

QX222 is highly sensitive to *par-1* RNAi while containing the CB4856 allele of *ppw-1* (see Table 1 and Table S2). Because both parents lose *par-1* RNAi sensitivity when either the CB4856 allele or the deletion allele of *ppw-1* is homozygous, we concluded that whatever is suppressing the insensitivity due to *ppw-1* in QX222 is multigenic and derived from a combination of alleles from both parents. To confirm this hypothesis and further test if the *ppw-1* suppression is recessive to the parent backgrounds, we looked at F1 from crosses of QX222 with CB4856 and NL3511. F1 from both crosses are highly insensitive (see Figure 2). Thus the alleles causing the *ppw-1* suppression are not found together in either parent and are recessive to both parent backgrounds.

### High level of complexity in response phenotypes

Our examination of QX217 and QX222 lead us to conclude that epistatic interactions of alleles derived from both parents are underlying the variation in *par-1* RNAi sensitivity for these RIAILs. The low frequency of these phenotypes among the RIAILs suggests that a large number of alleles may be involved. To confirm this complexity and rule out other explanations, such as spontaneous mutations that might have arisen during the making of the RIAILs, we looked at the segregation of sensitivity in a cross of QX217 and N2. From an assay of 182 F2s from this cross, we found a large excess of fully sensitive F2 (93%) over expectations for either a dominant (25%) or recessive (75%) insensitivity locus. The segregation of sensitivity suggests multiple alleles are required to act in a nonadditive manner to create the dominant insensitivity phenotype in QX217.

## Discussion

Our study extends previous work on the complexity of RNAi sensitivity in *C. elegans*. Previous work reported by Tijsterman *et al.* (2002) and Elvin *et al.* (2011) identified *ppw-1* as a major effect gene in RNAi sensitivity variation between N2 and CB4856, and also found F2 and recombinant inbred line evidence supporting complexity in the trait. Here we have focused on the dominance, number, and interactions of alleles affecting this trait.

### Interpretation of *par-1* RNAi assay

Our study examined natural variation in the germline embryonic lethality effects of *par-1* RNAi treatment of *C. elegans* strains N2 and CB4856. We interpret our results to reflect variation in the efficacy of the RNAi pathway in the germline.

We did not test the tissue specificity of our results. However, we assume that our results are specific to the germline because somatic RNAi has been reported to function similarly in N2 and CB4856 (Tijsterman *et al.* 2002).

Variation independent of the RNAi pathway has the potential to explain some of our results. Technical variation between RNAi plates, within and between assay days, likely added nongenetic noise to our results. Replicate RNAi plates of N2 and CB4856 had highly consistent measures of embryonic lethality (see Table S1), suggesting there was little noise in our embryonic lethality results near the extreme values (0% and 100%).

For genetic variation, we controlled for background levels of embryonic lethality in our strains using a mock RNAi control. However, at different levels of *par-1* knockdown, genes independent of RNAi may influence lethality. Thus, some genetic variation may be specific to the lethality phenotype. Also, some variation may be gene-specific, affecting the lethality of knocking down *par-1* but not necessarily all other essential genes. CB4856s lack of germline RNAi sensitivity has been demonstrated for many genes (Tijsterman *et al.* 2002; Elvin *et al.* 2011), suggesting our results may not be gene-specific. The degree to which our results were influenced by genetic variation specific to the embryonic lethality phenotype remains to be tested by examination of additional embryonic RNAi phenotypes.

### Complexity and epistasis

We strongly reject a single-locus model to explain the segregation of germline RNAi sensitivity between N2 and CB4856 but have not addressed how complex this trait is. The simplest model with *ppw-1*, *zeel-1/peel-1*, plus a single unlinked recessive allele that confers a complete loss of sensitivity can be rejected (χ^2^ test, *P* < 10^−100^). It would result in an accurate expectation for high sensitivity F2 but would produce an excess of low sensitivity F2 and would fail to explain the large intermediate sensitivity class. Under a model of strict additivity, additional loci would need to confer intermediate losses in sensitivity to explain our segregation results. A single unlinked recessive allele conferring an intermediate loss of sensitivity produces an expectation of 59.4% high sensitivity, 19.8% intermediate sensitivity, and 20.8% low sensitivity, nearly identical to our observed F2 proportions (χ^2^ test, *P* = 0.94). Thus an additive unlinked two-locus model is sufficient to explain our F2 observations but as we next discuss, is insufficient to explain our RIAIL results.

The class of 18 RIAILs with intermediate sensitivities confirms the complexity of the trait and provides additional information about the number of loci and the additivity of their effects. We observed four RIAILs that suggest sensitivity-decreasing effects of alleles independent of *ppw-1* and 14 RIAILs that suggest sensitivity-increasing epistatic effects of combinations of alleles. These two different effects alone would imply three or more alleles affecting sensitivities in addition to *ppw-1*.

What is a likely model for the *ppw-1*−independent sensitivity-decreasing effects? Additional loci could impair the RNAi response in the germline while not fully eliminating it. Only four of 121 of the RIAILs with N2 markers flanking *ppw-1* show this effect, a strong skew from the 50% or greater expectation for one or more additional additive loci. Linkage to *ppw-1* or *zeel-1*/*peel-1* could explain the frequency skew. Nonadditive interactions among a large number of loci (five or more) could also explain the frequency skew. The dominant epistatic action of QX217, which is one of these four RIAILs, suggests non-additivity may be the more likely explanation.

What can explain the 14 RIAILs with intermediate sensitivities and CB4856 markers flanking *ppw-1*? These RIAILS were completely unexpected because neither CB4856 nor N2 suppress the loss of sensitivity effects of *ppw-1*. From this result we can conclude that two or more loci are interacting epistatically to increase sensitivity. Considering that only one RIAIL (QX222) had high sensitivity and *ppw-1(CB4856)*, we can infer that either many more nonadditive loci are involved or that linkage among loci led to this rare combination of alleles. Either way, it is clear that three or more epistatically interacting loci are causing increased sensitivities in the presence of *ppw-1(CB4856)*.

In summary, the simplest model for variation in germline RNAi sensitivity between N2 and CB4856 involves *ppw-1*, three or more loci with sensitivity-increasing effects, and five or more loci with sensitivity-decreasing effects, all of which interact non-additively.

An outstanding question in genetics is how common this level of complexity and epistasis is (Phillips 2008). One parallel example of unexpected complexity is in variation in thermal tolerance in *C. elegans* (Gaertner *et al.* 2012). Similar to our findings, Gaertner *et al.* (2012) found strong suppression and enhancement effects between alleles. As high-throughput genotyping and phenotyping increase the breadth of traits that are studied, it will be interesting to see how large a role nonadditive effects play in natural variation.

### Identifying additional loci

Our study confirmed the established role of *ppw-1* in germline RNAi sensitivity variation in *C. elegans*. However, the additional causal loci remain to be characterized. Although epistasis challenges linkage analysis, there are feasible strategies for further dissecting these kinds of gene complexes.

One approach to identifying additional QTL is sequential elimination (Sinha *et al.* 2008; Lorenz and Cohen 2012), where additional recombinants are generated with the effects of known QTL controlled by crossing parents that are both homozygous for the known QTL. For example, our RIAIL QX222 could be crossed with CB4856 and could also be crossed with NL3511 (*ppw-1* deletion in N2 background with low sensitivity) to generate two recombinant populations, both of which would be homozygous for null alleles of *ppw-1*. These recombinant populations could then be used to map the loci that suppress the effects of *ppw-1* in QX222.

Another approach to isolate the effects of specific loci would be to generate congenic strains (also known as near isogenic lines) (Shao *et al.* 2010). For example, if linkage to *ppw-1* or the *zeel-1/peel-1* locus obscured causal loci on chromosome I in our QTL analysis, we could generate near isogenic lines with CB4856 segments added to the N2 background or N2 segments added to the CB4856 background to isolate the effects of these segments. This powerful approach could also be used to dissect large QTL identified from sequential elimination.

We hope that using these alternative mapping approaches will help dissect the functional molecular mechanisms underlying complex epistasis in RNAi sensitivity and will help resolve our generalized model of the genetic architecture of natural variation.

## Supplementary Material

Supporting Information
