# Supplementary material for: Resistance to Germline RNA Interference in a Caenorhabditis elegans Wild Isolate Exhibits Complexity and Nonadditivity
Source: G3 (Bethesda). 2013 Jun 1;3(6):941–7. doi: 10.1534/g3.113.005785 (PMC3689805; doi:10.1534/g3.113.005785)
Supplement: Supporting Information [file supp_3_6_941__index.html]

Resistance to Germline RNA Interference in a Caenorhabditis elegans Wild Isolate Exhibits Complexity and Nonadditivity — Supporting Information 

# Resistance to Germline RNA Interference in a *Caenorhabditis elegans* Wild Isolate Exhibits Complexity and Nonadditivity

## Supporting Information for Pollard and Rockman, 2013

**Files in this Data Supplement:**

- Supporting Information - Figure S1, File S1, and Tables S1-S2 (PDF, 259 KB)
- Figure S1 - (A) Expected F2 phenotypes considering *ppw-1* and *zeel-1/peel-1* segregation. (B) Proportion of F2s expected with total lethality (red), partial lethality (orange), and no lethality (green) as a function of q. (PDF, 189 KB)
- Table S1 - Counts of dead embryos and hatched larvae for individual N2, CB4856, and F1 (N2xCB4856) worms (PDF, 87 KB)
- Table S2 - Recombinant inbred advance intercross line (RIAIL) *par-1* induced lethality data (PDF, 135 KB)
- File S1 - Rqtl package input file for RIAIL mapping analysis (.txt, 439 KB)
