# Supplementary material for: Resistance to Germline RNA Interference in a Caenorhabditis elegans Wild Isolate Exhibits Complexity and Nonadditivity
Source: G3 (Bethesda). 2013 Jun 1;3(6):941–7. doi: 10.1534/g3.113.005785 (PMC3689805; doi:10.1534/g3.113.005785)
Supplement: Supporting Information [file supp_g3.113.005785_005785SI.pdf]

G3 · Genes | Genomes | Genetics

Resistance to germline RNAi in a *Caenorhabditis elegans* wild isolate exhibits complexity and non-additivity

Daniel A. Pollard*[1], Matthew V. Rockman*

*Department of Biology, New York University, New York, NY 10003
[1] Current Address: Division of Biological Sciences, University of California, San Diego, CA 92093

DOI: 10.1534/g3.113.005785

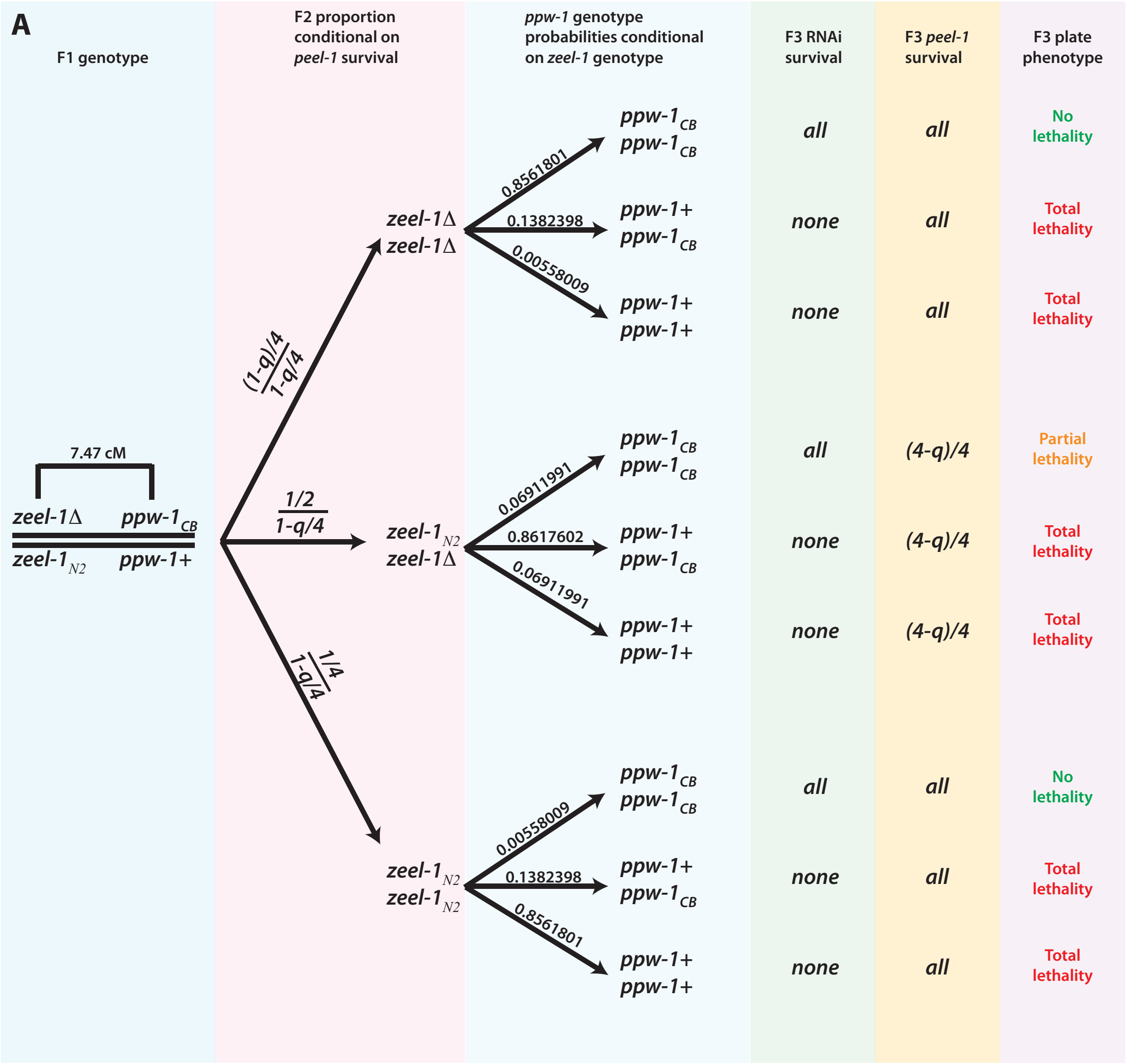
A
F1 genotype
F2 proportion conditional on peel-1 survival
ppw-1 genotype probabilities conditional on zeel-1 genotype
F3 RNAi survival
F3 peel-1 survival
F3 plate phenotype
7.47 cM
zeel-1Δ ppw-1CB
zeel-1N2 ppw-1+
(1-q)/4 / 1-q/4
1/2 / 1-q/4
1/4 / 1-q/4
zeel-1Δ zeel-1Δ
zeel-1N2 zeel-1Δ
zeel-1N2 zeel-1N2
0.8561801
0.1382398
0.00558009
0.06911991
0.8617602
0.06911991
0.00558009
0.1382398
0.8561801
ppw-1CB ppw-1CB
ppw-1+ ppw-1CB
ppw-1+ ppw-1+
all
none
(4-q)/4
No lethality
Total lethality
Partial lethality

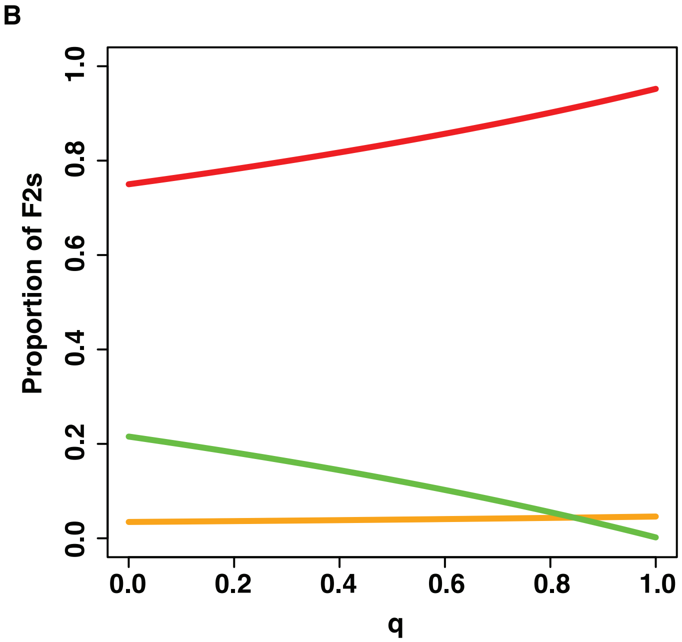
B
Proportion of F2s
q
0.0
0.2
0.4
0.6
0.8
1.0

Figure S1 (A) Expected F2 phenotypes considering *ppw-1* and *zeel-1/peel-1* segregation. The penetrance of *peel-1*-induced lethality among embryos homozygous for the CB4856 allele of *zeel-1* is given by the variable q. (B) Proportion of F2s expected with total lethality (red), partial lethality (orange), and no lethality (green) as a function of q.

File S1

Rqtl package input file for RIAIL mapping analysis.

File S1 is available for download at http://www.g3journal.org/lookup/suppl/doi:10.1534/g3.113.005785/-/DC1.

Table S1 Counts of dead embryos and hatched larvae for individual N2, CB4856, and F1 (N2xCB4856) worms.

Strain	Date	RNAi Vector	Dead Embryos	Larvae	Fraction Dead	Induced Lethality
N2	6/2/11	L4440	0	69	0.00	
N2	6/2/11	L4440	0	62	0.00	
N2	6/2/11	L4440	0	61	0.00	
N2	6/2/11	L4440	0	61	0.00	
N2	6/2/11	L4440	0	55	0.00	
N2	6/2/11	L4440	0	40	0.00	
N2	6/2/11	L4440	0	68	0.00	
N2	6/2/11	L4440	0	65	0.00	
N2	6/2/11	L4440	0	67	0.00	
N2	6/2/11	L4440	0	69	0.00	
N2	6/2/11	L4440	0	58	0.00	
N2	6/2/11	L4440	2	60	0.03	
N2	6/2/11	L4440	2	50	0.04	
**N2**	**Total**	**L4440**	**4**	**785**	**0.01**	
N2	6/2/11	par-1	39	0	1.00	1.00
N2	6/2/11	par-1	25	0	1.00	1.00
N2	6/2/11	par-1	57	0	1.00	1.00
N2	6/2/11	par-1	47	0	1.00	1.00
N2	6/2/11	par-1	24	0	1.00	1.00
N2	6/2/11	par-1	50	0	1.00	1.00
N2	6/2/11	par-1	60	0	1.00	1.00
N2	6/2/11	par-1	40	0	1.00	1.00
N2	6/2/11	par-1	65	0	1.00	1.00
N2	6/2/11	par-1	36	0	1.00	1.00
N2	6/2/11	par-1	44	0	1.00	1.00
N2	6/2/11	par-1	64	0	1.00	1.00
**N2**	**Total**	**par-1**	**551**	**0**	**1.00**	**1.00**
CB4856	6/2/11	L4440	0	58	0.00	
CB4856	6/2/11	L4440	0	56	0.00	
CB4856	6/2/11	L4440	1	54	0.02	
CB4856	6/2/11	L4440	1	51	0.02	
CB4856	6/2/11	L4440	1	51	0.02	
CB4856	6/2/11	L4440	1	38	0.03	
CB4856	6/2/11	L4440	1	24	0.04	
**CB4856**	**Total**	**L4440**	**5**	**332**	**0.01**	
CB4856	6/2/11	par-1	0	30	0.00	0.00
CB4856	6/2/11	par-1	0	53	0.00	0.00
CB4856	6/2/11	par-1	0	51	0.00	0.00
CB4856	6/2/11	par-1	0	33	0.00	0.00
CB4856	6/2/11	par-1	0	56	0.00	0.00
CB4856	6/2/11	par-1	0	50	0.00	0.00
CB4856	6/2/11	par-1	1	62	0.02	0.00
CB4856	6/2/11	par-1	1	33	0.03	0.01
**CB4856**	**Total**	**par-1**	**2**	**368**	**0.01**	**0.00**
F1	6/2/11	L4440	0	20	0.00	
F1	6/2/11	L4440	2	50	0.04	
F1	6/2/11	L4440	3	55	0.05	
F1	6/2/11	L4440	3	48	0.06	
F1	6/2/11	L4440	4	54	0.07	
F1	6/2/11	L4440	5	55	0.08	
F1	6/2/11	L4440	3	31	0.09	
F1	6/2/11	L4440	2	20	0.09	
F1	6/2/11	L4440	4	37	0.10	

**F1**	**Total**	**L4440**	**26**	**370**	**0.07**	
F1	6/2/11	par-1	12	1	0.92	0.92
F1	6/2/11	par-1	62	3	0.95	0.95
F1	6/2/11	par-1	60	0	1.00	1.00
F1	6/2/11	par-1	71	0	1.00	1.00
F1	6/2/11	par-1	37	0	1.00	1.00
F1	6/2/11	par-1	67	0	1.00	1.00
F1	6/2/11	par-1	20	0	1.00	1.00
F1	6/2/11	par-1	70	0	1.00	1.00
F1	6/2/11	par-1	70	0	1.00	1.00
F1	6/2/11	par-1	12	0	1.00	1.00
**F1**	**Total**	**par-1**	**481**	**4**	**0.99**	**0.99**

Table S2 Recombinant inbred advance intercross line (RIAIL) *par-1* induced lethality data. Counts of dead embryos and hatched larvae on *par-1* RNAi and L4440 control plates were used to calculate induced lethality levels for each RIAIL.

Strain (QX)	*par-1* dead embryos	*par-1* larvae	pL4440 dead embryos	pL4440 larvae	par-1 fraction dead	pL4440 fraction dead	Induced Lethality	ppw-1 5' SNP	ppw-1 3' SNP
4	163	0	18	43	1.00	0.30	1.00	N2	N2
6	213	0	15	127	1.00	0.11	1.00	N2	N2
8	22	0	2	7	1.00	0.22	1.00	N2	N2
11	40	0	6	17	1.00	0.26	1.00	N2	N2
13	859	23	8	591	0.97	0.01	0.97	N2	N2
14	76	0	6	61	1.00	0.09	1.00	N2	N2
17	223	0	6	260	1.00	0.02	1.00	N2	N2
18	104	0	93	8	1.00	0.92	1.00	N2	N2
19	10	0	4	42	1.00	0.09	1.00	N2	N2
20	35	0	1	27	1.00	0.04	1.00	N2	N2
22	59	0	2	33	1.00	0.06	1.00	N2	N2
24	17	24	4	36	0.41	0.10	0.35	N2	N2
25	13	0	3	60	1.00	0.05	1.00	N2	N2
27	11	0	0	94	1.00	0.00	1.00	N2	N2
29	49	0	9	20	1.00	0.31	1.00	N2	N2
30	58	0	1	70	1.00	0.01	1.00	N2	N2
31	36	0	6	41	1.00	0.13	1.00	N2	N2
32	3	0	3	9	1.00	0.25	1.00	N2	N2
33	33	0	16	82	1.00	0.16	1.00	N2	N2
34	58	0	1	14	1.00	0.07	1.00	N2	N2
37	6	0	0	7	1.00	0.00	1.00	N2	N2
39	105	0	5	72	1.00	0.06	1.00	N2	N2
41	28	0	2	61	1.00	0.03	1.00	N2	N2
43	2	0	3	7	1.00	0.30	1.00	N2	N2
47	13	0	4	20	1.00	0.17	1.00	N2	N2
51	52	0	11	74	1.00	0.13	1.00	N2	N2
52	9	0	8	3	1.00	0.73	1.00	N2	N2
54	49	0	12	29	1.00	0.29	1.00	N2	N2
61	58	0	15	33	1.00	0.31	1.00	N2	N2
64	420	325	9	464	0.56	0.02	0.56	N2	N2
66	189	0	13	62	1.00	0.17	1.00	N2	N2
68	146	0	65	28	1.00	0.70	1.00	N2	N2
69	39	0	1	15	1.00	0.06	1.00	N2	N2
71	10	0	0	12	1.00	0.00	1.00	N2	N2
73	192	0	2	142	1.00	0.01	1.00	N2	N2
78	101	0	0	40	1.00	0.00	1.00	N2	N2
79	53	0	33	7	1.00	0.83	1.00	N2	N2
80	95	0	7	97	1.00	0.07	1.00	N2	N2
82	188	0	28	107	1.00	0.21	1.00	N2	N2
83	57	0	3	69	1.00	0.04	1.00	N2	N2
84	38	0	0	42	1.00	0.00	1.00	N2	N2
85	131	0	9	88	1.00	0.09	1.00	N2	N2
87	34	0	8	31	1.00	0.21	1.00	N2	N2
90	6	0	2	21	1.00	0.09	1.00	N2	N2
91	54	0	1	76	1.00	0.01	1.00	N2	N2
92	121	0	24	65	1.00	0.27	1.00	N2	N2
96	5	0	2	15	1.00	0.12	1.00	N2	N2
97	37	0	3	56	1.00	0.05	1.00	N2	N2
98	127	0	13	55	1.00	0.19	1.00	N2	N2

102	3	0	3	13	1.00	0.19	1.00	N2	N2
103	6	0	2	4	1.00	0.33	1.00	N2	N2
104	170	0	47	132	1.00	0.26	1.00	N2	N2
105	20	0	4	66	1.00	0.06	1.00	N2	N2
106	55	0	15	28	1.00	0.35	1.00	N2	N2
110	35	0	15	39	1.00	0.28	1.00	N2	N2
112	48	0	55	4	1.00	0.93	1.00	N2	N2
114	120	0	18	145	1.00	0.11	1.00	N2	N2
116	123	0	2	169	1.00	0.01	1.00	N2	N2
117	130	0	12	106	1.00	0.10	1.00	N2	N2
120	239	0	13	299	1.00	0.04	1.00	N2	N2
121	112	0	82	15	1.00	0.85	1.00	N2	N2
122	250	0	9	45	1.00	0.17	1.00	N2	N2
124	41	0	2	9	1.00	0.18	1.00	N2	N2
125	135	0	6	106	1.00	0.05	1.00	N2	N2
128	68	0	1	57	1.00	0.02	1.00	N2	N2
129	40	0	6	29	1.00	0.17	1.00	N2	N2
132	43	0	22	87	1.00	0.20	1.00	N2	N2
136	131	0	1	81	1.00	0.01	1.00	N2	N2
137	133	0	3	150	1.00	0.02	1.00	N2	N2
140	97	0	10	166	1.00	0.06	1.00	N2	N2
143	52	0	6	11	1.00	0.35	1.00	N2	N2
144	2	0	5	3	1.00	0.63	1.00	N2	N2
148	126	0	0	253	1.00	0.00	1.00	N2	N2
151	141	0	33	68	1.00	0.33	1.00	N2	N2
152	184	0	16	107	1.00	0.13	1.00	N2	N2
155	150	0	29	59	1.00	0.33	1.00	N2	N2
156	95	0	37	78	1.00	0.32	1.00	N2	N2
157	110	0	10	66	1.00	0.13	1.00	N2	N2
159	96	0	8	101	1.00	0.07	1.00	N2	N2
160	24	0	3	37	1.00	0.08	1.00	N2	N2
161	21	0	3	98	1.00	0.03	1.00	N2	N2
163	10	0	0	3	1.00	0.00	1.00	N2	N2
164	168	0	13	65	1.00	0.17	1.00	N2	N2
165	36	0	6	4	1.00	0.60	1.00	N2	N2
170	91	0	9	100	1.00	0.08	1.00	N2	N2
173	53	0	4	17	1.00	0.19	1.00	N2	N2
174	6	0	7	20	1.00	0.26	1.00	N2	N2
175	71	0	45	8	1.00	0.85	1.00	N2	N2
178	78	0	37	60	1.00	0.38	1.00	N2	N2
181	109	0	8	198	1.00	0.04	1.00	N2	N2
182	172	0	8	119	1.00	0.06	1.00	N2	N2
183	38	0	0	85	1.00	0.00	1.00	N2	N2
184	102	0	2	109	1.00	0.02	1.00	N2	N2
185	117	0	1	142	1.00	0.01	1.00	N2	N2
188	92	0	15	65	1.00	0.19	1.00	N2	N2
189	8	0	1	20	1.00	0.05	1.00	N2	N2
190	91	0	0	98	1.00	0.00	1.00	N2	N2
191	17	0	17	56	1.00	0.23	1.00	N2	N2
192	29	0	6	38	1.00	0.14	1.00	N2	N2
193	4	0	0	24	1.00	0.00	1.00	N2	N2
195	40	0	1	8	1.00	0.11	1.00	N2	N2
203	196	0	2	155	1.00	0.01	1.00	N2	N2
204	128	0	35	80	1.00	0.30	1.00	N2	N2
206	15	0	8	23	1.00	0.26	1.00	N2	N2
207	2	0	9	5	1.00	0.64	1.00	N2	N2
212	42	0	6	80	1.00	0.07	1.00	N2	N2
213	106	0	36	46	1.00	0.44	1.00	N2	N2

216	126	0	8	24	1.00	0.25	1.00	N2	N2
217	122	536	4	118	0.19	0.03	0.16	N2	N2
221	198	0	4	266	1.00	0.01	1.00	N2	N2
223	153	0	2	106	1.00	0.02	1.00	N2	N2
225	63	0	37	4	1.00	0.90	1.00	N2	N2
226	5	0	2	19	1.00	0.10	1.00	N2	N2
227	82	0	0	130	1.00	0.00	1.00	N2	N2
228	108	0	5	113	1.00	0.04	1.00	N2	N2
229	86	0	26	56	1.00	0.32	1.00	N2	N2
230	4	0	0	57	1.00	0.00	1.00	N2	N2
232	67	0	0	12	1.00	0.00	1.00	N2	N2
234	34	0	4	88	1.00	0.04	1.00	N2	N2
235	21	0	27	26	1.00	0.51	1.00	N2	N2
237	116	0	16	150	1.00	0.10	1.00	N2	N2
38	23	0	0	37	1.00	0.00	1.00	N2	CB4856
171	4	0	1	4	1.00	0.20	1.00	N2	CB4856
172	125	0	5	164	1.00	0.03	1.00	N2	CB4856
1	22	681	2	276	0.03	0.01	0.02	CB4856	CB4856
5	13	832	13	557	0.02	0.02	0.00	CB4856	CB4856
7	20	139	2	113	0.13	0.02	0.11	CB4856	CB4856
42	3	752	3	356	0.00	0.01	0.00	CB4856	CB4856
56	4	582	1	246	0.01	0.00	0.00	CB4856	CB4856
57	8	497	9	596	0.02	0.01	0.00	CB4856	CB4856
62	0	618	3	388	0.00	0.01	0.00	CB4856	CB4856
63	8	662	11	398	0.01	0.03	0.00	CB4856	CB4856
72	3	725	2	283	0.00	0.01	0.00	CB4856	CB4856
81	11	660	6	246	0.02	0.02	0.00	CB4856	CB4856
86	0	321	12	256	0.00	0.04	0.00	CB4856	CB4856
107	2	961	7	269	0.00	0.03	0.00	CB4856	CB4856
113	2	474	0	256	0.00	0.00	0.00	CB4856	CB4856
115	11	482	0	247	0.02	0.00	0.02	CB4856	CB4856
127	1	89	0	28	0.01	0.00	0.01	CB4856	CB4856
133	0	217	2	56	0.00	0.03	0.00	CB4856	CB4856
135	4	501	2	141	0.01	0.01	0.00	CB4856	CB4856
145	6	296	1	61	0.02	0.02	0.00	CB4856	CB4856
158	15	130	4	142	0.10	0.03	0.08	CB4856	CB4856
168	18	115	1	42	0.14	0.02	0.11	CB4856	CB4856
169	2	362	0	145	0.01	0.00	0.01	CB4856	CB4856
218	12	210	3	131	0.05	0.02	0.03	CB4856	CB4856
219	47	432	21	167	0.10	0.11	0.00	CB4856	CB4856
222	226	6	3	137	0.97	0.02	0.97	CB4856	CB4856
236	4	495	1	249	0.01	0.00	0.00	CB4856	CB4856
