# Supplementary material for: Resistance to Germline RNA Interference in a Caenorhabditis elegans Wild Isolate Exhibits Complexity and Nonadditivity
Source: G3 (Bethesda). 2013 Jun 1;3(6):941–7. doi: 10.1534/g3.113.005785 (PMC3689805; doi:10.1534/g3.113.005785)
Supplement: Supporting Information [file supp_g3.113.005785_FigureS1.pdf]

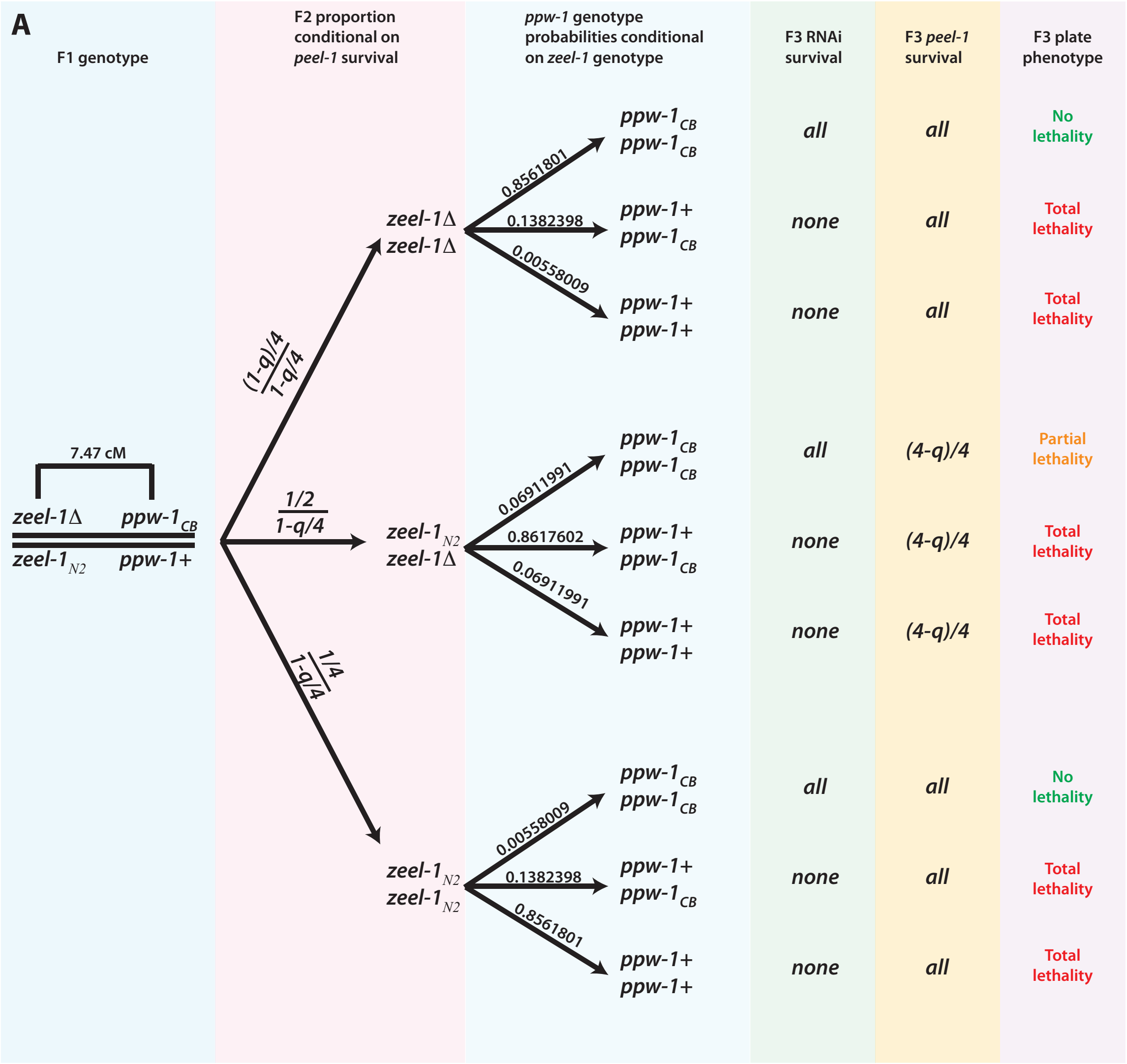
A
F1 genotype
F2 proportion conditional on peel-1 survival
ppw-1 genotype probabilities conditional on zeel-1 genotype
F3 RNAi survival
F3 peel-1 survival
F3 plate phenotype
7.47 cM
zeel-1Δ ppw-1CB
zeel-1N2 ppw-1+
(1-q)/4 / 1-q/4
1/2 / 1-q/4
1/4 / 1-q/4
zeel-1Δ zeel-1Δ
zeel-1N2 zeel-1Δ
zeel-1N2 zeel-1N2
0.8561801
0.1382398
0.00558009
0.06911991
0.8617602
0.06911991
0.00558009
0.1382398
0.8561801
ppw-1CB ppw-1CB
ppw-1+ ppw-1CB
ppw-1+ ppw-1+
all
none
(4-q)/4
No lethality
Total lethality
Partial lethality

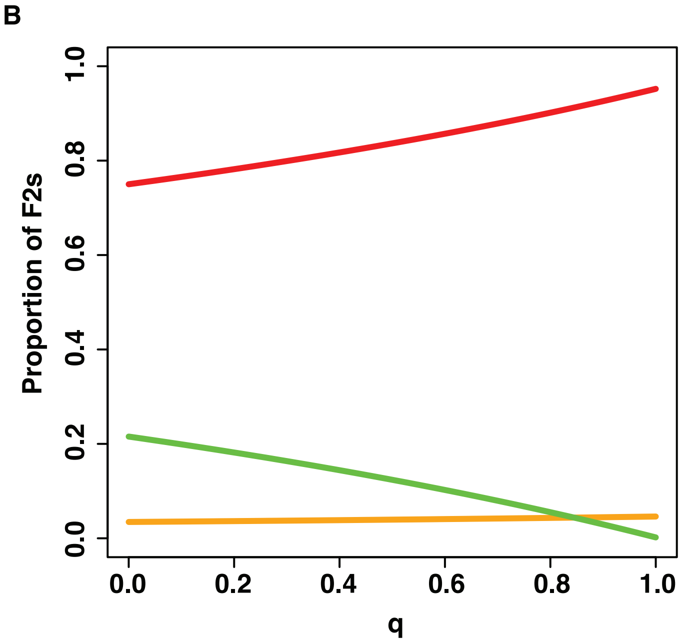
B
Proportion of F2s
q
0.0
0.2
0.4
0.6
0.8
1.0

Figure S1 (A) Expected F2 phenotypes considering *ppw-1* and *zeel-1/peel-1* segregation. The penetrance of *peel-1*-induced lethality among embryos homozygous for the CB4856 allele of *zeel-1* is given by the variable q. (B) Proportion of F2s expected with total lethality (red), partial lethality (orange), and no lethality (green) as a function of q.
