# Supplementary material for: Resistance to Germline RNA Interference in a Caenorhabditis elegans Wild Isolate Exhibits Complexity and Nonadditivity
Source: G3 (Bethesda). 2013 Jun 1;3(6):941–7. doi: 10.1534/g3.113.005785 (PMC3689805; doi:10.1534/g3.113.005785)
Supplement: Supporting Information [file supp_g3.113.005785_TableS1.pdf]

Table S1 Counts of dead embryos and hatched larvae for individual N2, CB4856, and F1 (N2xCB4856) worms.

Strain	Date	RNAi Vector	Dead Embryos	Larvae	Fraction Dead	Induced Lethality
N2	6/2/11	L4440	0	69	0.00	
N2	6/2/11	L4440	0	62	0.00	
N2	6/2/11	L4440	0	61	0.00	
N2	6/2/11	L4440	0	61	0.00	
N2	6/2/11	L4440	0	55	0.00	
N2	6/2/11	L4440	0	40	0.00	
N2	6/2/11	L4440	0	68	0.00	
N2	6/2/11	L4440	0	65	0.00	
N2	6/2/11	L4440	0	67	0.00	
N2	6/2/11	L4440	0	69	0.00	
N2	6/2/11	L4440	0	58	0.00	
N2	6/2/11	L4440	2	60	0.03	
N2	6/2/11	L4440	2	50	0.04	
**N2**	**Total**	**L4440**	**4**	**785**	**0.01**	
N2	6/2/11	par-1	39	0	1.00	1.00
N2	6/2/11	par-1	25	0	1.00	1.00
N2	6/2/11	par-1	57	0	1.00	1.00
N2	6/2/11	par-1	47	0	1.00	1.00
N2	6/2/11	par-1	24	0	1.00	1.00
N2	6/2/11	par-1	50	0	1.00	1.00
N2	6/2/11	par-1	60	0	1.00	1.00
N2	6/2/11	par-1	40	0	1.00	1.00
N2	6/2/11	par-1	65	0	1.00	1.00
N2	6/2/11	par-1	36	0	1.00	1.00
N2	6/2/11	par-1	44	0	1.00	1.00
N2	6/2/11	par-1	64	0	1.00	1.00
**N2**	**Total**	**par-1**	**551**	**0**	**1.00**	**1.00**
CB4856	6/2/11	L4440	0	58	0.00	
CB4856	6/2/11	L4440	0	56	0.00	
CB4856	6/2/11	L4440	1	54	0.02	
CB4856	6/2/11	L4440	1	51	0.02	
CB4856	6/2/11	L4440	1	51	0.02	
CB4856	6/2/11	L4440	1	38	0.03	
CB4856	6/2/11	L4440	1	24	0.04	
**CB4856**	**Total**	**L4440**	**5**	**332**	**0.01**	
CB4856	6/2/11	par-1	0	30	0.00	0.00
CB4856	6/2/11	par-1	0	53	0.00	0.00
CB4856	6/2/11	par-1	0	51	0.00	0.00
CB4856	6/2/11	par-1	0	33	0.00	0.00
CB4856	6/2/11	par-1	0	56	0.00	0.00
CB4856	6/2/11	par-1	0	50	0.00	0.00
CB4856	6/2/11	par-1	1	62	0.02	0.00
CB4856	6/2/11	par-1	1	33	0.03	0.01
**CB4856**	**Total**	**par-1**	**2**	**368**	**0.01**	**0.00**
F1	6/2/11	L4440	0	20	0.00	
F1	6/2/11	L4440	2	50	0.04	
F1	6/2/11	L4440	3	55	0.05	
F1	6/2/11	L4440	3	48	0.06	
F1	6/2/11	L4440	4	54	0.07	
F1	6/2/11	L4440	5	55	0.08	
F1	6/2/11	L4440	3	31	0.09	
F1	6/2/11	L4440	2	20	0.09	
F1	6/2/11	L4440	4	37	0.10	

**F1**	**Total**	**L4440**	**26**	**370**	**0.07**	
F1	6/2/11	par-1	12	1	0.92	0.92
F1	6/2/11	par-1	62	3	0.95	0.95
F1	6/2/11	par-1	60	0	1.00	1.00
F1	6/2/11	par-1	71	0	1.00	1.00
F1	6/2/11	par-1	37	0	1.00	1.00
F1	6/2/11	par-1	67	0	1.00	1.00
F1	6/2/11	par-1	20	0	1.00	1.00
F1	6/2/11	par-1	70	0	1.00	1.00
F1	6/2/11	par-1	70	0	1.00	1.00
F1	6/2/11	par-1	12	0	1.00	1.00
**F1**	**Total**	**par-1**	**481**	**4**	**0.99**	**0.99**
